# Some Thoughts Suggested by the Austro-Hungarian Problem

**Published:** 1914-09-15

**Authors:** Dorothy Richardson


### SOME THOUGHTS SUGGESTED BY THE AUSTRO-HUNGARIAN PROBLEM.

BY DOROTHY RICHARDSON.

Those who have followed the recent developments of the situation in Austro-Hungary will find Dr. Zsigmondy's ardent elaboration of the case of "Stomatology" particularly interesting at the present juncture. For the benefit of those who have not come to close quarters with the Austro-Hungarian situation we may state here that until comparatively recently dentistry in Austria and in Hungary has been carried on almost exclusively by the trained handicraftsman—the dental technician. Without "medical" training, either on its academic or its practical side, he has gone forth (legally accredited) from his highly specialized technical institutions, and has sufficed for all the simple dental needs of the public. For something like half a century the medical faculty has laid a gradually increasing siege upon the dental profession as represented by these trained technicians, claiming with ever-increasing firmness of conviction the recognition of the medical factor in dental operations. Finally, the Austrian Medical Chamber succeeded in persuading the Government that the filling and extracting of teeth are medical operations and cannot safely be left in the hands of those who have had no medical training. The Austrian Government, dentally profane, embodied this truism in an enactment to the effect that the technicians must keep to their workshops and that operative dentistry must be carried on by doctors alone.

Into the fabric of Austro-Hungarian statecraft was woven the fact that dentistry is not mechanics. No sooner, however, was dentistry safely transferred to the hands of the medical faculty than it appeared that dentistry is not "medicine"— that the fundamental quality in the dentist, the highly-specialized technique of dental operations is not to be gained by the processes that lead to the acquisition of a medical degree. An extraordinary *impasse* has been created. The technicians are legally disqualified. The medico-technical synthesis is not yet made. Dentistry, as we know it, does not yet, if we except a small number of foreign-trained practitioners and the few doctors who have specialized as stomatologists, exist in Austro-Hungary. The dental technician, trained and certificated, member of an ancient guild with the easily available rudiments of scientific knowledge at his disposal and the instructive experiences of dental practice in his hands, still holds his own. Theoretically legally banned, he enjoys the confidence of the public, and, very generally, the patronage of local magistrates. Many are the instances cited of the failure of prosecutions of dental technicians undertaken in provincial courts. The magistracy behaves, complains Dr. Gruen, in his recent appeal to the Government, "as though *clause* 343 did not exist, or if they are aware of it, do not recognize the filling and extracting of teeth as medical activities."

Over against the technicians are the stomatologists ("doctors" with dental qualifications and practicing dentistry), the dentally unqualified doctors who carry on dentistry with the aid of certified dental technicians (who are, of course, legally speaking, unqualified assistants), and the dentally unqualified doctors who employ assistants who are both legally and actually unqualified, barbers' assistants and other casual persons.

Actions brought against such doctors as these last by the Guild of Dental Technicians fail on the ground that dentistry is a medical occupation.

The most recent move on the part of medical Austria has been a formal appeal to the Government for protection against the "encroaching laity," under which category "Nature curers," "physical educationists" and "dental technicians" are accorded a special condemnation.

Taking all the circumstances into consideration it is not surprising that there should be in Austro-Hungarian professional circles an active body of stomatologists, of men who consider dentistry as a specialized branch of medicine, and only those worthy of the title dentist who have first qualified medically and then turned their attention to the special problems of dentistry.

Nor is it less than we should expect, that from this body, in the person of Dr. Zsigmondy, should come the ablest response that has yet appeared to the claim being put forward in Germany for a degree of Doctor of Dentistry on the ground that dental science has been built up by "dentists" and that dentistry is essentially different from medicine in material, in diagnostic and in therapeutics. The essence of Dr. Zsigmondy's response is first a satisfying demonstration, from the facts of dental history, to the effect that all the advances in dental science (mechanics excepted) have come from those dentists equipped with the medical degree, and secondly, that the dentist must, before he can avail to the advancement of his profession, learn to "think medically" in the fullest sense of the term.

Such a response, coming from the heart of the Austro-Hungarian complex, is peculiarly significant.

Particularly interesting to the onlooker is the proviso "mechanics excepted"—with the suggestion therein contained that the developments of dentistry on the medical side are relatively secondary.

The demand of the "dentists" for an absolute separation of "dentistry" from "medicine" is perhaps in its way as one-sided as the insistence that medicine is the greater half of dentistry. We may admit the essential practical distinctions all along the line between medicine and dentistry, we may grant the importance on the other hand, to the dentist of thinking medically, but we cnanot see that either the "dentist" or the "stomatologist" gets to the root of the situation. Still less can we find in the stomatological solution, the idea of dental training following on a complete medical course any way out of the Austro Hungarian situation. Freely admitting that history demonstrates beyond a doubt that "dentists" who have "thought medically" are the men who have made "dental science," we are, then, immediately faced with the question as to how much dental science on its "medical" side has contributed to the solution of the problem of dental caries and we would invite the stomatologists to look once more at the historical phenomenon of dentistry with this second consideration in view.

The "dental consciousness," the specialized thought of that body of persons whose main occupation has been the care of the teeth, dates, very obviously, from the moment of the invasion of the serene world of the dental handicraftsman by the developing complexities of medical science.

Nevertheless, behind the final product of that invasion, behind the highly individualized modern dentist the handicraftsman is still there. No knowledge, however complete, of the range of sciences that go to the making of the "doctor," on medical thought or medical practice will, even with dental training thrown in, make a dentist of the man in whom the essential gift of the handicraftsman, the mechanical sense, is absent. The best "dentists" are those in whom this gift, there at the outset, has been exquisitely trained, disciplined and developed. Thinking mechanically is at least as important to the dentist as thinking medically.

A timely confirmation of this fact is the *form* of the first original contribution by dentistry to the theory of disease. This original contribution, this first specifically *dental* 'generalization takes the form of a mechanical theorem. 'Medical" sciences, bacteriology, chemistry, histology, pathology, etc., have achieved in the domain of dentistry nothing more than "descriptions" of dental caries. "Mechanics" has thrown at last some light on its "cause." It is, of course, difficult to emphasize this essential "truth" without appearing to desire to belittle the influence of the various sciences which have helped to lead dentistry forward to its own special illumination. Such a desire would be absurd. The inter-action, the mutual inter-dependence of all the sciences is the condition of advance of every one of them. Their mutual relationships and indebtedness can no more be accurately estimated and apportioned than can water be cut with a fork. The fact remains, however, that whatever the contributary illuminations, it is from dentistry in its mechanical aspect that the truth has come forth. One of its first effects is an implicit criticism of "medical" and "surgical" dentistry, as they have so far existed.

Our recognition of the fact that diseases of the teeth are largely due to mechanical interference with the normal functions of the jaws by means of artificial feeding, thumb-sucking (and kindred bad habits), the consumption of soft, sweet foods stimulating and developing the sucking parts of the mouth and the centre of the jaw and leaving the sides unexercised, by the unwholesome affectation of the small mouthfull which makes mastication almost impossible—reveals the laborious occupations of dentistry in its medical and surgical aspects as largely negative. Humane and necessary though they be, they may, not inaptly, be compared to the building of a large and well-equipped hospital at the foot of a precipice over which the public is perpetually falling for want of a steering rail.

We are not, here, concerned with the fact that medicine is w thin her own territory well on the way to a confession of the futility of tinkering with symptoms, but, as we remarked in an earlier paper, medical dentistry as compared with general medicine is, in being faced with symptoms that are relatively static, in the better position to realize this futility.

Nor is it, of course, suggested that the mechanical truth about the teeth, the restatement of the phenomenon with which dental science is perpetually faced in terms of mechanical function is the whole truth about the teeth. Manifestly it is not, since the question of nutrition and the preservation of harmony are there at the outset. But this truth is a fundamental truth, an ultimate, something there is no getting behind—and it implies a revolution in dental practice.

It seems possible that before any practical settlement shall have been reached in Austro-Hungary, before the battle between technicians, "dentists" and stomatologists shall have been fought to a finish, dentistry itself will have quietly entered on a fresh phase of existence. The prospect of a universal public dental service opens the door to a national prophylactic dentistry—a possibility of interference at aetiological moments from infancy to maturity, a steering clear, particularly during infancy and early childhood, from the evil "habits" which interfere with the normal use and development of the jaws and teeth; (the difficulty of restoring the norm once it is upset and the still greater difficulty of retaining the restored norm are to be read in the records of the orthodontists).

The result of a generation of rational interference, in the light of the trugh revealed by the discovery of the mechanical factor in dental caries, would be primarily an enormous reduction in the incidence of that disease, and in the second place a transformation in the function of dentistry, which would become very largely educational, a matter of skilled supervision and advice.

Meanwhile it would appear that in Austro-Hungary, as elsewhere, the combatants within dental territory, the technicians, the "dentists" and the stomatologists might find a point of union in working towards their common recognition and organization as component parts of a public health body, a various body of technicians—versed in all the sciences which bear upon their craft, a little three-winged army of architects, the auxiliary architects of the human jaws and teeth.—*Dental Record.*

